# Novel exploration of 3D printed personalized total elbow arthroplasty to solve the severe bone defect after internal fixation failure of comminuted distal humerus fracture: A case report

**DOI:** 10.1097/MD.0000000000021481

**Published:** 2020-07-31

**Authors:** Naichao Wu, Shan Li, Yang Liu, Aobo Zhang, Bingpeng Chen, Qing Han, Jincheng Wang

**Affiliations:** a Department of Orthopedics; b Department of Dermatological, Second Hospital of Jilin University, Changchun, Jilin Province, China.

**Keywords:** bone defect, 3-dimensional printed prosthesis, internal fixation failure, preoperative simulation, total elbow arthroplasty

## Abstract

**Rationale:**

Severe bone defect could often occur after removing the fractured fixation plates of comminuted fracture in the distal humerus. The reoperation of internal fixation or conventional total elbow arthroplasty could hardly restore the anatomy structure and function of the elbow. However, a novel exploration of 3-dimensional (3D) printed personalized elbow prosthesis was presented in this work. This is a rare and successful treatment for the severe bone defect after removing the fractured fixation plates of comminuted distal humerus fracture.

**Patient concerns:**

A 60-year-old male patient was admitted to the hospital with the chief complaint of right elbow joint pain and limitation of motion for 10 years. He suffered from an open reduction with internal fixation surgery 10 years ago due to a fall injury-induced right distal humerus fracture.

**Diagnoses:**

Plain radiographs and computed tomography scan revealed fracture lines, fracture displacement, and fixator breakage in the right distal humerus. Pain, swelling, and limitation of motion could be found in the physical examination. Fixation failure and nonunion after internal fixation of comminuted distal humerus fracture were considered.

**Interventions:**

The patient was treated with 3D printed personalized TEA and functional rehabilitation exercises.

**Outcomes:**

No severe complications were observed during the 36 months follow-up. The patient could complete the daily activities without pain. The hospital for special surgery score increased from 15 points before surgery to 90 points 36 months after surgery.

**Lessons:**

The 3D printed personalized prosthesis could successfully reconstruct the anatomical structures and function of the elbow joint with a severe bone defect. The 3D printed personalized total elbow arthroplasty might provide a feasible method for treating the complex elbow joint diseases in the elderly.

## 1 Introduction

Complex distal humerus fractures often cause severe joint surface damage. With the development of surgical approaches, the progress has gradually improved the rehabilitation of the elbow joint after internal fixation.^[1]^ However, the complex comminuted fracture's treatment effect is still not satisfactory enough after open reduction and internal fixation (ORIF) surgery. Some complications such as joint pain, joint stiffness, motion limitation, and even internal fixation failure for some patients in the short- or long-term follow-ups, affect the patient's primary elbow joint activity and reduce the quality of life.^[2–5]^ Conventional total elbow arthroplasty (TEA) is an effective treatment for some of the complex distal humerus fractures without apparent bone defect, which can significantly reduce postoperative complications. Besides, the TEA has the advantages of the short operation time, high functional score, and better early dysfunction score compared with ORIF.^[6–9]^ However, the conventional solutions could not restore the anatomy structure and function in the cases with a severe bone defect; this is because that no anatomical complementary augment for the bone defect was provided in the conventional prosthesis. At present, with the prosperity of 3-dimensional (3D) printing technology and development of medical-engineering interaction, preoperative simulation, accurate intraoperative navigation, and personalized prosthesis design can be performed on such complicated cases with the severe bone defect.^[10]^ Therefore, we presented a case of solving the severe bone defect after internal fixation failure of comminuted distal humerus fracture by 3D printed personalized TEA. The patient has provided informed consent for publication of the case. 3D printing technology and its related ideas were throughout the whole process of the preoperative simulation, design of elbow arthroplasty, manufacturing of bone models, and design of personalized elbow joint prosthesis.

## 2 Ethics

In this study, 3D printing individualized elbow prosthesis implantation was approved by the Medical Ethics Committee of the Second Hospital of Jilin University. Changchun, Jilin Province, China (2015), Research and Inspection No. (189). The patient and his family agreed to the surgical plan and signed the informed consent form.

## 3 Case report

### 3.1 Patient characteristics

The patient is a male, 60 years old with a height of 178 cm, and a weight of 77 kg. He was admitted to hospital for complaint of joint pain and limitation of motion for nearly 10 years. He had a history of ORIF surgery 10 years ago due to a fall injury-induced right distal humerus fracture.

**Figure 1:**
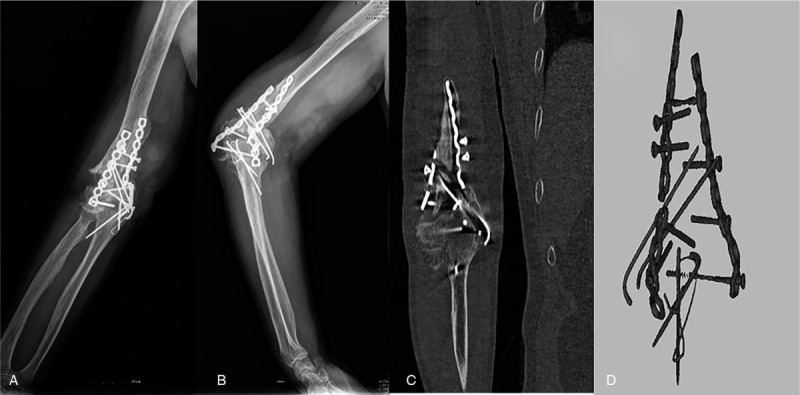
Preoperative image of a 60-year-old male patient diagnosed as fixation failure and nonunion after internal fixation of comminuted distal humerus fracture. (A) Anteroposterior plain radiograph of the right elbow joint. (B) Lateral plain radiographs of the right elbow joint. (C) Computed tomography image of the right elbow joint. (D) Reconstruction of the fractured plate by Mimics.

**Figure 2:**
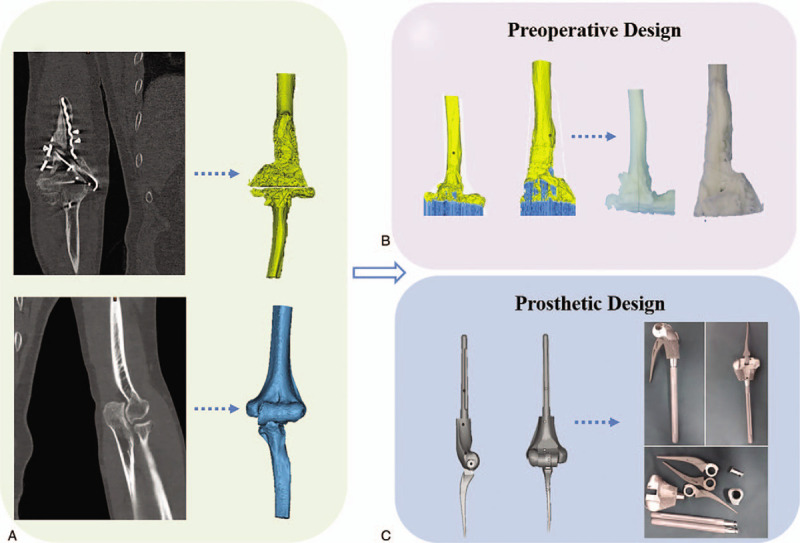
The process diagram of the preoperative design and prosthesis design. (A) Three-dimensional reconstruction of the bilateral elbow joint. (B) Preoperative design by the right elbow joint resin model. (C) Prosthesis design with the contralateral mirror images and manufacturing personalized prosthesis with Titanium alloy.

Physical examination displayed that the right elbow joint was swollen, the pain was obvious during the activity, and the range of motion was limited (motion arc <30°, rotation range <30°). There was no evident obstacle in the right hand; the skin feeling in the right upper limb was reduced. The elbow joint function evaluation was poor (hospital for special surgery [HSS] score 15 points, Mayo score 55 points). Plain radiographs, computed tomography (CT) scan, and 3D reconstruction of the plates by Mimics 20.0 software (Materialise, Leuven, Belgium) revealed fracture lines, fracture displacement, and fixator breakage in the right distal humerus (Fig. 1). The personalized TEA was decided after consultation and discussion. The process diagram of the preoperative and prosthesis design is shown in Figure 2.

### 3.2 Preoperative design

#### 3.2.1 3D reconstruction of the bilateral elbow joint

The CT was scanned by Philips company's 256-row spiral CT (Philips, Amsterdam, Netherlands). The parameters are as follows: tube current 232 mA, tube voltage 120 kV, and scanning layer thickness 1 mm. After scanning, the CT data of the bilateral elbow joints in digital imaging and communications in medicine format was collected and imported into Mimics software (Materialise) for 3D reconstruction of the bilateral elbow joints, and the stereolithography (STL) format file was saved. The STL was a format of triangular mesh to represent the 3D CAD model.

#### 3.2.2 Manufacture of the resin elbow joint model by stereo lithography apparatus (SLA) technology

The STL format file of the 3D right elbow joint model was imported into Magics software (Materialise). The model should be repaired without structural errors and moved to the appropriate position of workbench. The support structure must be generated to prepare for printing. At last, the adjusted STL file was exported to the SLA450 resin printer, and the patient's stereolithography resin elbow joint model was printed. The resin models prepared in this process could provide references for the osteotomy range and preoperative simulation.

#### 3.2.3 Design and manufacture of the personalized elbow prosthesis

The 3D printed hinge elbow joint prosthesis was designed according to the mirror image of the symmetrical humerus. The personalized elbow joint prosthesis was modeled on the blueprint of typical Coonrad–Morrey elbow prosthesis. According to the affected bone condition of the humerus and the residual bone morphology after osteotomy, the prosthesis stem's length and size were determined. For insurance purposes, we designed three “sleeves” with thicknesses of 4, 8, and 12 mm, which were used to compensate for the size deviation between the osteotomy and the prosthesis. The elbow joint prosthesis was fabricated with Ti6Al4V powder using electron beam melting techniques in an Arcam Q10 plus metal printer.

#### 3.2.4 Preoperative simulation for matching test

The resin model of the right elbow joint was used to simulate intraoperative osteotomy. The affected bone of the proximal ulna and the distal humerus was removed, then the 3D printed personalized prosthesis was implanted into the residual bone. The simulation process verified that the prosthesis matched well with bone.

### 3.3 Surgical procedure

**Figure 3:**
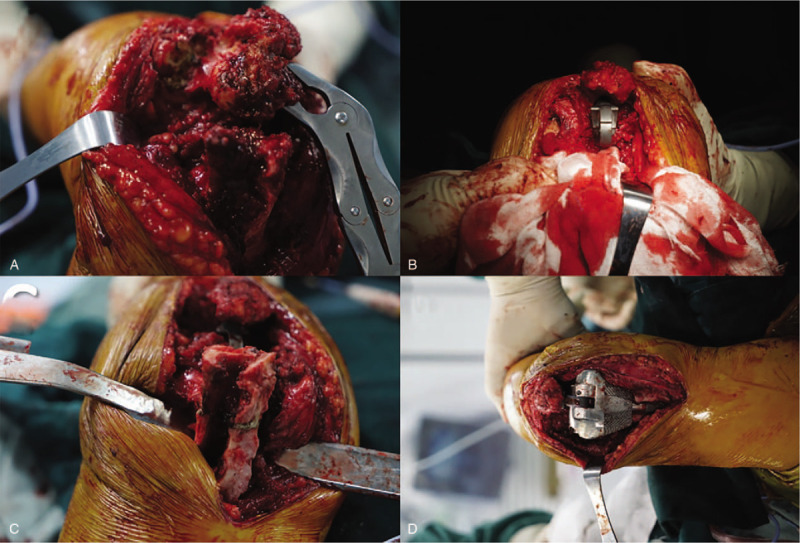
The total elbow arthroplasty surgical procedure. (A) Exposure of the ulnar end and the osteotomy. (B) Installation of ulnar end prosthesis. (C) Exposure of the humeral end and the osteotomy. (D) Installation of humeral end prosthesis and the assembly.

The patient was taken the supine position after general anesthesia and disinfection. The medial incision of the right elbow was taken. First, the obsolete fixation device was removed, and the osteotomy was performed as simulated. The broken bone at the proximal end of the ulna, and the olecranon were cut according to the preoperative design; then the olecranon tip was removed. The elbow was flexed to separate the distal end of the humerus from the ulna. The middle part of the trochlea humerus was removed by the bone saw, and the medullary cavity of the humerus was opened. Then the T-handle was used to insert into the medullary cavity before testing the prosthesis. The prosthesis edge was just flush with the capitulum humerus and the articular surface of humeral supracondylar. The ulna was reamed along the long axis, the proximal ulna and the distal humerus were prepared, and the prosthesis was inserted into the ulnar and the humeral medullary cavity, respectively. The bolts were used to fix the prosthesis at both ends. The prosthesis was ensured that elbow joints could realize flexible extension and flexion, and the motion arc of the passive elbow joint was ensured to reach 140° nearly. Besides, to maintain the humerus’ integrity as much as possible, wire fixation was used to bound the large bone pieces at the distal end of the humerus. Finally, the intraoperative positioning showed that the prosthesis was in the correct position; the incision was closed layer by layer. (The surgical procedure is shown in Fig. 3)

### 3.4 Outcomes and follow-up

**Figure 4:**
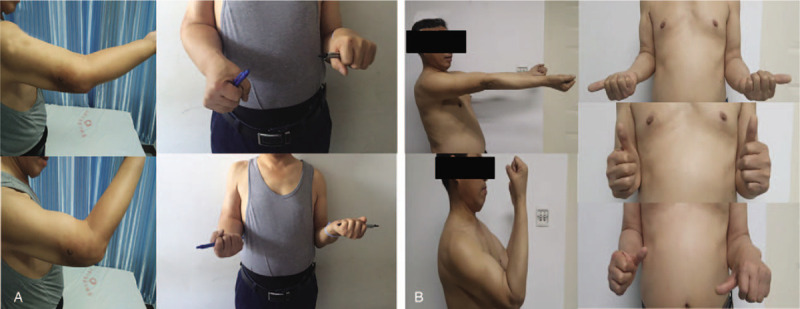
The motion range (straightening, bending, pronation, and supination) before (A) and after (B) surgery (36th month) of the right elbow.

**Figure 5:**
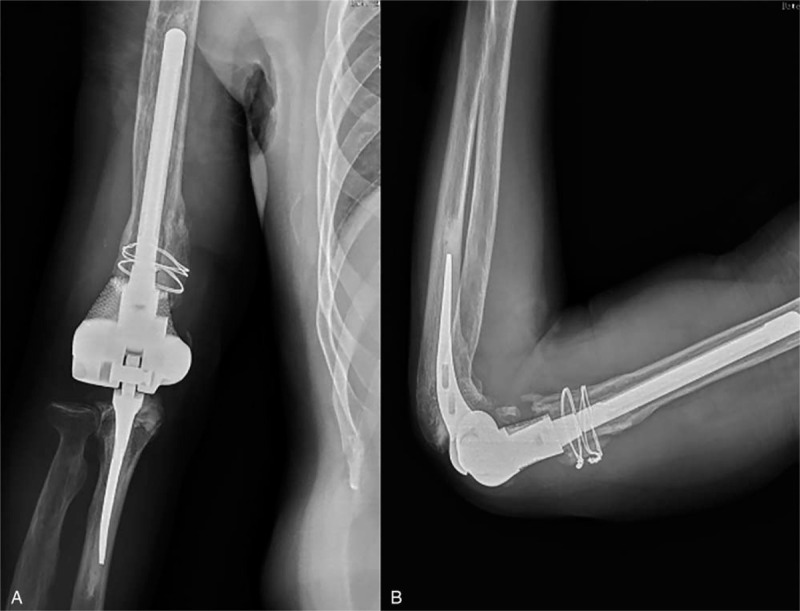
Plain radiographs in the 36 months follow-up. (A) Anteroposterior plain radiograph of the right elbow joint. (B) Lateral plain radiographs of the right elbow joint.

The operation was completed successfully. It took 65 minutes from osteotomy to the installation of the personalized elbow prosthesis. The blood loss volume was 235 mL. Rehabilitation guidance was given in the following rehabilitation exercise. The elbow function was evaluated with Mayo and HSS function scores during the follow-up, as shown in Table 1. The motion ranges before and after surgery (36th months) of the right elbow are shown in Figure 4. The elbow joint motion range increased from 15-45° to 0-135°, and the rotation range reached 90°. The Mayo score was 95 points, and the HSS score was 90 points. The elbow joint plain radiographs at the 1st, 3rd, 6th, 12th, 24th, and 36th months after operation were reviewed. The plain radiographs of last time (36th months after operation) showed in Figure 5 revealed that the prosthesis was in a good position and no sign of loosening. No complications such as fractures and dislocations around the joints were found. There was no symptom of nerve damage according to the physical examination. There was no sign of infection according to the laboratory test results of erythrocyte sedimentation rate and C-reactive protein. The patient was sufficient to complete the basic life activities such as washing, dressing, and eating.

**Table 1:**
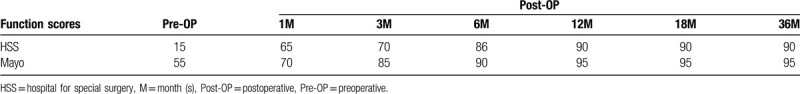
Hospital for special surgery and Mayo scores during the follow-up.

## 4 Discussion

The usual treatment for the complex distal humerus fractures was ORIF surgery. In recent years, as for the old patients, elbow joint arthroplasty gradually got the favor of doctors and patients. Indications for elbow joint arthroplasty are rheumatoid arthritis involving elbow joints, various inflammatory joint diseases, old complex elbow joint fractures, elbow joint stiffness, nonunion, congenital malformations, and related tumor treatment.^[11]^ Kamineni and Morrey^[12]^ proposed the indications of TEA for the treatment of distal humerus fractures: age >65 years; no need for vigorous activity; severe osteoporosis; and comminuted fracture or the fracture which is difficult to reset and fixed. Zha et al^[13]^ suggested that the patient age in developing countries could relax to >60 years. A multicenter, prospective, randomized, controlled trial showed that TEA was a preferred alternative for ORIF in elderly patients with complex distal humeral fractures who were not amenable to stable fixation.^[14]^ For the patient with failure internal fixation, it was difficult to get stable fixation with the reoperation of ORIF, especially the patient with a severe bone defect and diminution of bone mineral quality.^[15]^ The TEA could achieve a better therapeutic effect than the ORIF group in short-term and medium-term efficacy, especially for 1st-time ORIF failures.^[16]^ Some research indicated that there was no significant difference between primary TEA surgery and surgery after failed internal fixation.^[17]^ As for this case, the fractured fixation plates should be removed, and noticeable bone loss in the right elbow joint was found. TEA was considered to be operated. While severe bone defect would happen in this patient because of removing the failed fixation, conventional TEA might not restore the integrity of anatomy and function. So, 3D printing personalized prosthesis was selected to reconstruct the anatomy structure for this patient.

In this case, 2 main problems were involved in restoring the structure and function of the elbow joint. The 1st was to determine the scope of osteotomy at the distal of the humerus after removing the failed fixation plate; the 2nd was how to design the personalized prosthesis. To determine the scope of osteotomy, the 3D reconstruction technology and stereolithography resin model were adopted. The stereolithography appearance was proven to be reliable and precise in diagnosing and treating some complex orthopedic diseases.^[18]^ In this case, the 3D reconstruction from the CT scan data and the 3D printed stereolithography resin humerus model were used to estimate the osteotomy scope. In the personalized prosthesis design procedure, it was crucial to restoring the primary anatomy structure of the affected humerus. Some studies indicated that there were no significant differences between the left and right humerus in any of the measurements performed.^[19]^ The mirror image of the unaffected humerus was used to design the personalized prosthesis based on the Coonrad–Morrey prosthesis. The Coonrad–Morrey prosthesis is a semi-restricted hinge prosthesis and one of the most widely used prostheses in the United States.^[20]^ It has been found in Guoshen's^[21]^ study that the carrying angle of upper limbs was generally smaller than normal (even varus), and many Chinese patients have obvious convex on the outside of proximal forearms after the TEA with Coonrad–Morrey prosthesis. However, the design of 3D printed personalized prosthesis was modeled entirely according to the mirror structure of the unaffected side, retaining the carrying angle of the humeral shaft and the anterior angle of the humerus. The diameters of the humeral prosthesis stem were designed by the parameters of the affected humerus marrow cavity. The personalized prosthesis could ensure the best contact between the prosthesis and the bone to ensure the prosthesis stabilization. The preoperative simulation experiment of assembling personalized prosthesis with the residual stereolithography humerus after osteotomy was operated to ensure the prosthesis's applicability. Although the short-term follow-up showed satisfactory outcomes, more clinical cases and longer-term follow-up are needed to prove the clinical efficacy of this novel exploration.

## 5 Conclusion

This case demonstrated that 3D printing technology could assist in the preoperative design and the manufacture of personalized elbow prosthesis for TEA. The personalized elbow prosthesis could complete the anatomy and function reconstruction of the elbow joint. We provided a feasible therapy to solve the severe bone defect after internal fixation failure of comminuted distal humerus fracture by 3D printed personalized TEA.

## Acknowledgment

The authors are grateful to Dr Kesong Zhang and Hao Chen for their kind assistance with this article.

## Author contributions

**Conceptualization:** Jincheng Wang.

**Data curation:** Shan Li, Yang Liu.

**Methodology:** Bingpeng Chen.

**Software:** Aobo Zhang.

**Writing – original draft:** Naichao Wu, Qing Han.

**Writing – review & editing:** Yang Liu, Qing Han.
